# Microarray-Based Serotyping and Molecular Characterization of Virulence and Antimicrobial Resistance of *Salmonella enterica* from Swine Meat Samples in Abattoirs and Wet Markets of Metro Manila, Philippines

**DOI:** 10.3390/foods15020187

**Published:** 2026-01-06

**Authors:** Rance Derrick N. Pavon, Jonah Feliza B. Mora, Michael Joseph M. Nagpala, Abbie Codia, Homer D. Pantua, Windell L. Rivera

**Affiliations:** 1 Pathogen-Host-Environment Interactions Research Laboratory, Institute of Biology, College of Science, University of the Philippines Diliman, Quezon City 1101, Metro Manila, Philippines; rnpavon@up.edu.ph (R.D.N.P.); jbmora1@up.edu.ph (J.F.B.M.); mmnagpala@up.edu.ph (M.J.M.N.); 2 BioAssets Corporation, City of Santo Tomas 4234, Batangas, Philippines; abbie.codia@bioassets.com.ph (A.C.); homer.pantua@bioassets.com.ph (H.D.P.)

**Keywords:** antimicrobial resistance, Check & Trace *Salmonella*, Microarray serotyping, *Salmonella enterica*, serovars, virulence genes

## Abstract

*Salmonella* is a globally prevalent and diverse group of pathogenic bacteria that reside in food animals, such as swine. They possess transmissible antimicrobial resistance (AMR) and virulence factors, causing outbreaks with varying disease outcomes. This study identified and characterized 110 *Salmonella enterica* isolates from swine meat in abattoirs and wet markets of Metro Manila, Philippines. Thirteen different *S. enterica* serovars were identified using the Check & Trace microarray platform. The most prevalent were Rissen, Typhimurium 1, 4, [5], 12:i:-, Anatum, and Derby. This study is also the first to report serovar Soerenga in the Philippines and Asia. A high prevalence of virulence genes was observed, namely, *hilA* (75.45%), *avrA* (73.64%), *mgtC* (72.73%), *pipB* (66.36%), *sseC* (58.18%), and *spi4R* (53.64%), with no plasmid-borne *spvC* and *spvR*. A high prevalence of *bla*_TEM_ (44.55%) was also observed, consistent with the phenotypic AMR profiles. Additionally, 14.81% of the isolates exhibited multidrug resistance. Statistical associations and predictions were also found among virulence genes, serovars, and location types, which highlight implications of *Salmonella* contamination and serovar variations. These findings suggest the need for continuous surveillance of *Salmonella*, especially for emerging or rare serovars, the deeper investigation of virulence and AMR mechanisms, and improved regulation and sanitation throughout food animal industries.

## 1. Introduction

*Salmonella* is a foodborne, pathogenic, and Gram-negative bacterium classified into two species, namely *S. enterica* and *S. bongori*. *S. enterica* is divided into six subspecies comprising around 3000 known serovars [1]. Humans often become infected with *Salmonella* through the consumption of contaminated food of animal and plant origin, as well as contact with infected domestic animals [2,3]. Infections result in serovar-specific pathologies, whether typhoidal (e.g., Typhi), which manifest as mild to severe fevers, or nontyphoidal (e.g., Typhimurium and Enteritidis), which primarily cause self-limiting diarrhea [2]. *Salmonella* is also considered a critical priority pathogen with alarming resistance rates to third-generation cephalosporins, carbapenems, and fluoroquinolones [4]. This pathogen also carries diverse virulence genes, which are clustered in the currently identified 24 *Salmonella* pathogenicity islands (SPIs), contributing to host cell adhesion, invasion, and survival mechanisms [5].

Accurate tools for detection and serovar identification are crucial for the surveillance of *Salmonella*, which remains a global public health concern. However, the gold standard for *S. enterica* serotyping remains serological testing under the Kauffmann–White–Le Minor scheme (KWL), which relies on the discrimination of 46 O-antigens, 114 H-antigens, and typhoidal serovar-specific Vi-antigens [6,7,8,9]. Unfortunately, this requires the storage of more than 250 antisera and 350 antigens, is laborious and time-consuming, subjective and complicated to interpret, with tendencies for cross-reactivity and variable antigenic expression [7,9,10,11]. Molecular tools, such as the polymerase chain reaction (PCR), enabled faster and more sensitive serotyping of *S. enterica* O- and H-antigen-associated genes. However, complex banding patterns can occasionally prevent precise identification of rare serovars or lead to cross-reactivity [12,13]. Meanwhile, whole-genome sequencing (WGS) possesses greater discriminatory and informative power, although it remains costly, complicated, and requires bioinformatic expertise [14]. Alternatively, DNA microarrays rely on the hybridization of complementary probes to post-amplified target DNA for rapid, accurate, and high-throughput gene detection for applications such as identification, subtyping, characterization, and expression quantification [15]. Check & Trace *Salmonella* (CTS) is a microarray-based method developed and commercialized by Check-Points B.V. in Wageningen, the Netherlands, that generates serovar-discriminatory patterns from PCR amplification products without the need for complex bioinformatic analyses. Serovars that remain uncharacterized in this platform are labeled as genovars [16].

In the Philippines, the swine industry is a significant contributor to food animal production, accounting for approximately 14% of the agricultural value from 2000 to 2020, with a trend of increasing growth [17]. A high prevalence of *S. enterica* has been reported in slaughtered swine and wet market meats in Metro Manila, Philippines, suggesting potential threats to producers and consumers, as well as significant impacts on production yields [13,18,19,20]. However, most previous studies in the country relied only on multiplex PCR banding patterns, which require further validation through the KWL scheme. Therefore, this study used microarray technology to determine the serovar identities of *Salmonella* isolates collected from swine samples from wet markets and slaughterhouses. Isolates were then characterized and associated with their genotypic virulence patterns. Additionally, their genotypic and phenotypic AMR profiles were determined and corroborated.

## 2. Materials and Methods

### 2.1. Revival and Recovery of S. enterica Isolates

A total of one hundred and ten (*n* = 110) *S. enterica* isolates were previously isolated in 2018–2019 from abattoirs (*n* = 70) and wet markets (*n* = 40) and stored in glycerol stocks at −20 °C at the Pathogen-Host-Environment Interactions Research Laboratory, Institute of Biology, College of Science, University of the Philippines Diliman. Isolates were obtained from various swine meat samples from wet markets and tonsil and jejunum samples from slaughterhouses in Metro Manila, Philippines. Ethical review and approval were waived for this study due to informed consent obtained from the Philippine National Meat Inspection Service. Animal slaughter and evisceration were performed according to national regulations. Informed consent was also obtained from veterinarians in charge of the abattoirs for sample collection. For the current study, isolates were subjected to revival and recovery using standard procedures with some modifications [13,21]. Initially, 100 μL of glycerol stock cultures were inoculated into 900 μL of Trypticase Soy Broth (TSB) (Becton, Dickinson and Company, Franklin Lakes, NJ, USA), followed by incubation at 37 °C for 18–24 h. For isolation, loopfuls of TSB cultures were streaked onto Xylose Lysine Deoxycholate (XLD) agar plates (Becton, Dickinson and Company, NJ, USA), followed by incubation at 37 °C for 18–24 h. Presumptive *S. enterica* isolates (black colonies on red agar media) were then transferred and purified on Trypticase Soy agar (TSA) (Becton, Dickinson and Company, NJ, USA) for downstream processes.

### 2.2. DNA Extraction

The boil-lysis method was used for DNA extraction, following standard protocols [13,18]. Colonies from 18- to 24 h old *S. enterica* cultures were suspended in 50 μL of 1X Tris-EDTA (TE) buffer, followed by boiling at 100 °C for 10 min, and then cooled. This is followed by centrifugation at 2656× *g* for 5 min. Supernatants were then collected and stored at −20 °C for subsequent confirmatory and characterization assays.

### 2.3. PCR Confirmation of Salmonella enterica

To confirm the identity of *S. enterica*, DNA extracts were subjected to *invA* gene PCR, following the protocols and primers well-established in previous studies [20,22,23]. All reactions had a total volume of 12.5 μL, consisting of 6.25 μL of 2X GoTaq^®^ Green Master Mix (Promega, Madison, WI, USA), 0.5 μL each of *invA* gene primers [22] at 10 μM concentrations, 4.25 μL of nuclease-free water, and 1 μL of DNA template. *Salmonella enterica* subsp. *enterica* ATCC^®^ (American Type Culture Collection) serovar Typhimurium (ATCC 14028^TM^) via Kwik-Stik^TM^ (Microbiologics, St Cloud, MN, USA) was used as the positive control. More information regarding all genes investigated in this study is presented in Table 1, while PCR protocols can be found in Table 2.

### 2.4. Microarray-Based Salmonella enterica Serotyping

To identify serovars, Check & Trace *Salmonella* (CTS) (Check-Points B.V., Wageningen, The Netherland), a World Organization for Animal Health (OIE registration number: 20110106) and Association of Official Analytical Collaboration International (AOAC license number: 121001) validated and certified DNA microarray-based kit, was used following the manufacturer’s instructions. A single colony from 18–24 h cultures of confirmed *S. enterica* was pierced by a colony sampler, followed by suspension in 100 μL lysis buffer and heating at 99 °C for 15 min for DNA extraction. DNA extracts were then subjected to several DNA recognition steps through PCR, followed by serovar determination using the ArrayTube™ DNA microarray platform and Check-Points™ software version 4.11.0.72.

### 2.5. Detection of Virulence and Antimicrobial Resistance Genes

Eight representative chromosomal virulence genes from *Salmonella* pathogenicity islands (SPIs) 1–5, namely, *avrA*, *hilA*, *sseC*, *mgtC*, *spi4R*, and *pipB*, were analyzed alongside two plasmid-borne genes, *spvC* and *spvR*, and three β-lactam resistance genes, *bla*_TEM_, *bla*_CTX-M_, and *bla*_SHV_ using previously optimized protocols and conditions [23,31]. A total of 110 confirmed *S. enterica* isolates were screened for these genes. Each virulence multiplex PCR reaction was 12.5 μL in volume, consisting of 6.25 μL of 5X MyTaq™ HS Red Mix (Bioline, London, UK), 0.25 μL each of 10 µM primers, 2 μL of DNA template, and nuclease-free water to complete the total volume. Singleplex PCR reactions followed the same composition, except for *spi4R*, which used primer concentrations of 20 μM. For multiplex PCR for *bla* genes, we performed 12.5 μL reactions comprising 6.25 μL of 5× MyTaq HS Red Mix (Bioline), 0.25 μL of each primer (*bla*_TEM_ at 10 μM, *bla*_CTX-M_, and *bla*_SHV_ at 30 μM), 2 μL of DNA template, and nuclease-free water to make up the total volume.

Singleplex and multiplex PCR primer details are in Table 1 while PCR conditions can be found in Table 2. For virulence gene controls, Kwik-Stik^TM^ (Microbiologics) kits were used, involving ATCC *S. enterica* serovars Typhimurium (14028^TM^) and Enteritidis (13076^TM^) for *avrA*, *sseC*, *mgtC*, *pipB*, and *spi4R*, and serovar Choleraesuis (7001^TM^) for *hilA*, *spvC*, and *spvR*. For *bla* gene controls, previously confirmed *bla*_TEM-_ and *bla*_CTX-M_-positive *S. enterica* isolates in the laboratory [21], and ATCC *Klebsiella pneumoniae* (700603^TM^) for *bla*_SHV_ were used.

### 2.6. PCR Product Visualization

Following amplification, all PCR products were subjected to electrophoresis on 2% agarose gels (Vivantis, Subang Jaya, Malaysia) containing 1x Gel Red^®^ DNA Gel Stain (Biotium, Fremont, CA, USA). Five microliters of each PCR product were loaded into individual wells, accompanied by a 100 bp Bioline Hyperladder™ DNA molecular weight marker (Meridian Bioscience, MEM, Cincinnati, OH, USA) for accurate estimation of product sizes. Electrophoretic separation was performed using a CBS Scientific gel electrophoresis system (Thermo Fisher Scientific, Waltham, MA, USA), with a 1× Tris-Acetate-EDTA (TAE) running buffer, at 280 V for 45 min. Visualization of the PCR products was achieved through a Vilber Lourmat gel documentation system (Vilber, Collégien, France).

### 2.7. Antimicrobial Susceptibility Testing

Antimicrobial susceptibility testing was performed using the automated VITEK^®^ 2 system (bioMérieux, Marcy-l’Étoile, France) and its proprietary GN70 cards following the manufacturer’s instructions. A total of 12 antimicrobials, namely, ampicillin (AMP), ampicillin/sulbactam (SAM), piperacillin/tazobactam (TZP), ceftriaxone (CRO), cefepime (FEP), aztreonam (ATM), ertapenem (ETP), meropenem (MEM), amikacin (AMK), gentamicin (GEN), tobramycin (TOB), and trimethoprim/sulfamethoxazole (SXT), were tested and interpreted. The latest breakpoints (version 15.0 for 2025) for minimum inhibitory concentrations (MICs) of antimicrobials under Enterobacterales were based on the European Committee on Antimicrobial Susceptibility Testing (EUCAST) to determine susceptibility at standard dosage (S) and resistance (R). Briefly, three to four colonies of fresh NA cultures of *S. enterica* isolates were suspended in 0.45% sterile saline solution using sterile cotton swabs with an adjusted density of 0.5 McFarland measured using their proprietary DensiChek™ device (bioMérieux, Marcy-l’Étoile, France). Following this, suspensions were loaded into cassettes and into the VITEK^®^ 2 system for card-filling and sealing before being transferred into the incubation (35.5 °C) and analysis chamber for up to 18 h with automatic visualization and analysis every 15 min to reveal MICs for manual interpretation.

### 2.8. Data Analysis

Excel Office 365 (Microsoft) was used to analyze and visualize serovar, virulence, and AMR data, and generate graphs and heat maps. Meanwhile, the Flourish© visualization platform (Canva, London, UK) was used to generate tree maps for serovar distributions. SPSS Build 1.0.0.1447 (IBM, Armonk, NY, USA) was used for statistical analyses. Descriptive statistical analysis, particularly Fisher’s exact test, was used to determine significant associations between virulence genes and to evaluate the correlation between genotypic and phenotypic AMR data. Meanwhile, binary logistic regression was used to determine whether specific *S. enterica* serovars (independent variables) can predict location type, virulence, and *bla* gene presence (dependent variables), thereby determining potential pathogenicity and AMR variations across serovars. Odds ratios, *p*-values, and 95% confidence intervals (CI) were determined to signify the predictor effects on location type and the prevalence of *bla* and virulence genes. Statistical significance of all analyses was based on a *p*-value less than 0.05.

## 3. Results

### 3.1. Distribution of Salmonella enterica Serovars

**Figure 1:**
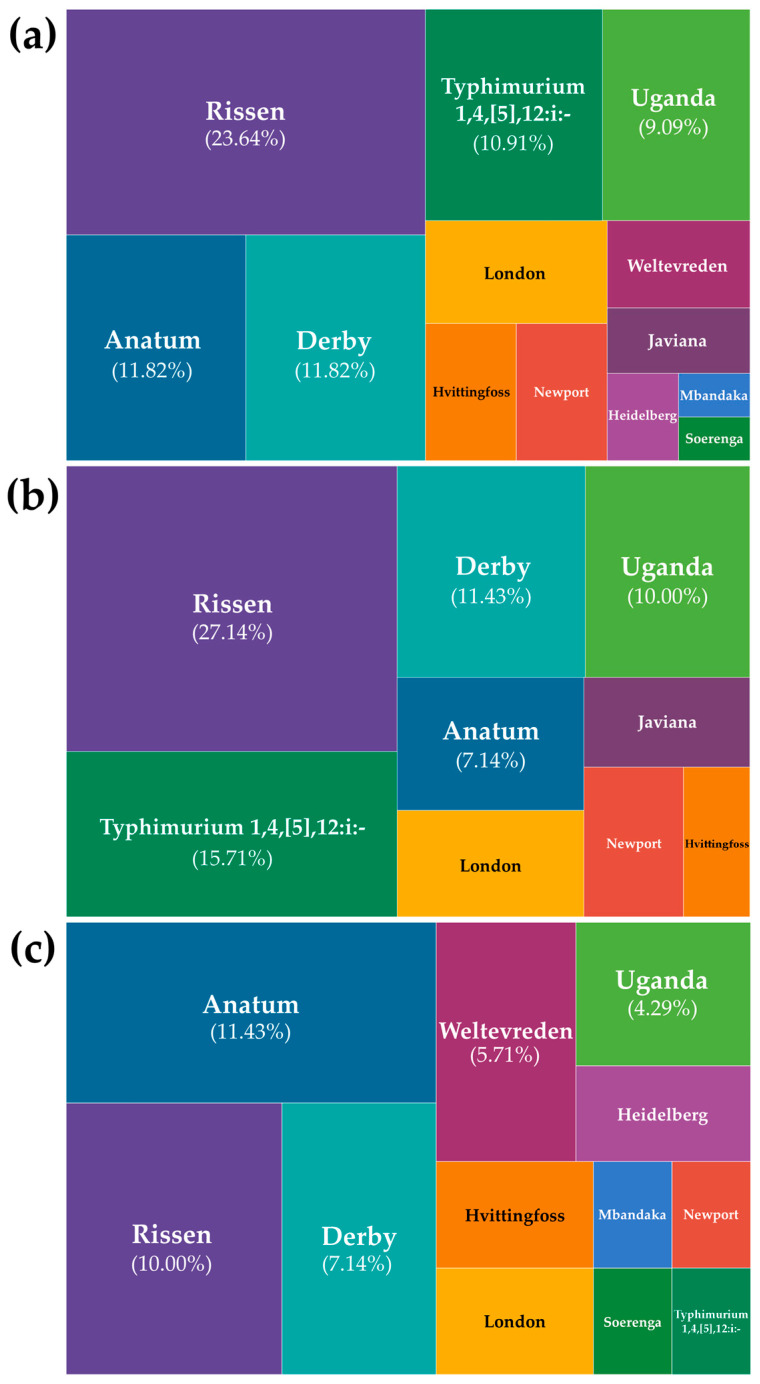
Tree maps showing the relative distributions and frequency variations of Salmonella *enterica* subsp. *enterica* serovars from swine samples in Metro Manila, Philippines, among (**a**) all 110 isolates; (**b**) abattoir isolates (*n* = 70); and (**c**) wet market isolates (*n* = 40).

All 110 isolates were subjected to microarray-based serotyping through CTS, which successfully classified 90% (99/110) of *S. enterica* into 13 known serovars. However, 10% (11/110) were unassigned due to incomplete or no prediction, and were instead designated as genovar codes. As shown in Figure 1a, the five most prevalent *S. enterica* serovars were Rissen (23.64%, 26/110), followed by both Anatum and Derby (11.82%, 13/110), monophasic variant Typhimurium 1, 4, [5], 12:i:- (10.91%, 12/110), and Uganda (9.09%, 10/110), with this study, being the first to report serovar Soerenga (0.91%, 1/110) in the Philippines and in Asia, isolated from a raw pork chop sample from a wet market in Manila.

Variations in serovar prevalence across location types (abattoirs and wet markets) were also observed (Figure 1b,c), although statistical analysis and frequencies may be affected by the difference in sample size between the two location types. Typhimurium 1, 4, [5], 12:i:- was the second most prevalent serovar among abattoir isolates at 15.71% (11/70), while it was found in only 1.43% (1/40) of wet market isolates. In contrast, Anatum was the most prevalent serovar among wet market isolates at 11.43% (8/40), while Rissen was the most prevalent serovar (27.14%, 19/70) among abattoir isolates, with Anatum only at 7.14% (5/70).

Statistical analysis using binary logistic regression to determine whether serovars can predict location types (abattoirs and wet markets), with serovar Rissen as the reference category due to its high prevalence and relevance among other *Salmonella* serovars in the global swine industry as well as being the most detected in the current study, and excluding genovars from the analysis, showed that only one out of 13 serovars was significant (*p* < 0.05). Serovar Anatum was observed to be 4.343 times more likely to be found in wet markets than in abattoirs, relative to serovar Rissen (*p*-value = 0.042; odds ratio = 4.343; 95% CI: 1.056–17.860). Interestingly, serovar Typhimurium 1, 4, [5], 12:i:-, although it showed less frequency in wet markets than abattoirs, was not significant (*p* > 0.05) in regression analysis (*p*-value = 0.217; odds ratio = 0.247). It is also worth noting that differences in isolate counts from abattoirs (*n* = 70) and wet markets (*n* = 40) may also affect regression analysis, resulting in wider 95% confidence intervals.

### 3.2. Frequency of AMR and Virulence Genes

**Figure 2:**
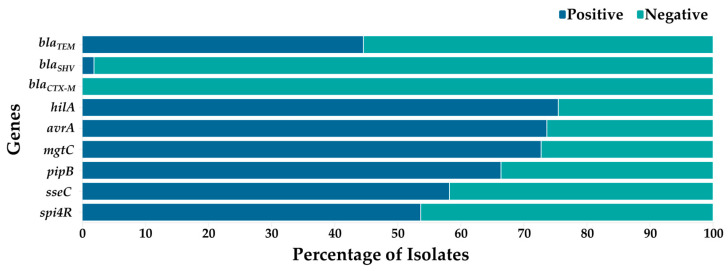
Prevalence of AMR and virulence genes among *S. enterica* isolates.

Variations in AMR and virulence gene prevalence were observed among *Salmonella* isolates (Figure 2). For AMR genes, the most prevalent was *bla*_TEM_ at 44.55%, followed by *bla*_SHV_ at 1.82%, with no positive isolates for *bla*_CTX-M_. Meanwhile, for the eight virulence genes detected within SPIs 1 to 5, the most prevalent was *hilA* (75.45%), followed by *avrA* (73.64%), *mgtC* (72.73%), *pipB* (66.36%), *sseC* (58.18%), and *spi4R* (53.64%), with no positive isolates for plasmid virulence genes *spvC* and *spvR*.

Fisher’s exact test among 15 virulence gene pairs, excluding *spvC* and *spvR*, revealed nine significant associations (*p* < 0.05, two-sided), namely, *avrA* with *mgtC*, *pipB*, and *sseC*, *mgtC* with *hilA* and *pipB*, *pipB* with *hilA* and *spi4R*, and lastly, *sseC* with *mgtC* and *pipB* (Table 3).

### 3.3. Variations in Gene Prevalence Among Salmonella enterica Serovars

**Figure 3:**
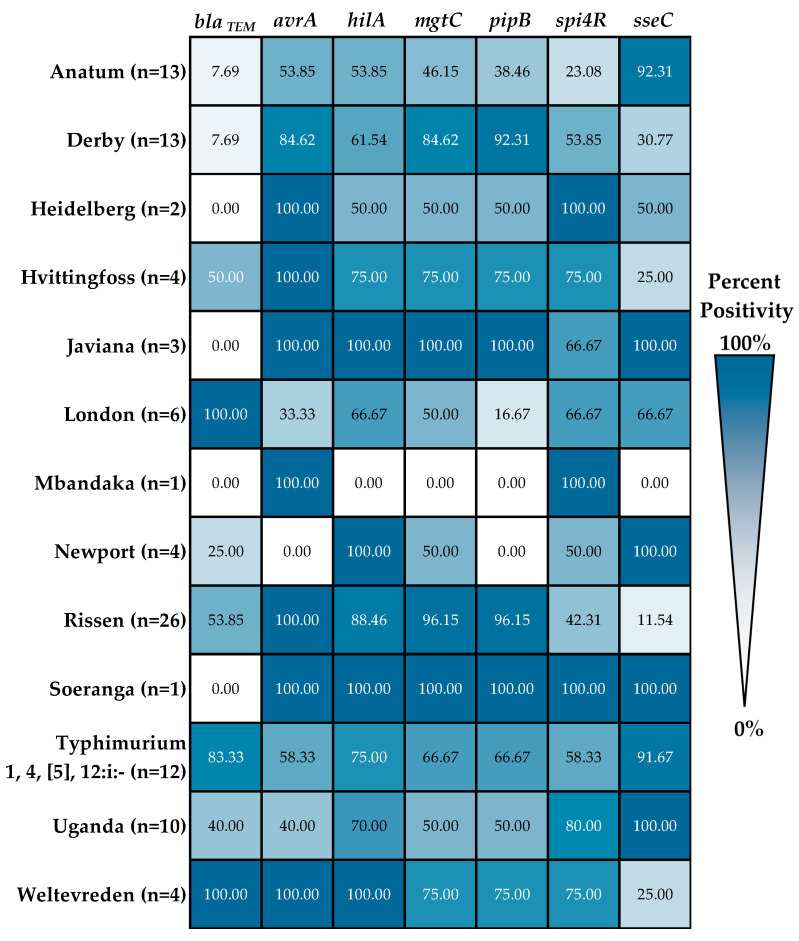
Heatmap of *bla* and virulence genes prevalence among known *S. enterica* serovars generated using Microsoft Office 365 Excel software. Darker shades on the heatmap indicate a higher positivity rate, while lighter shades approaching white indicate lower to no positivity.

*S. enterica* serovars in this study possessed varying AMR and virulence gene prevalence, although these numbers may also be affected by isolate counts. Since no isolates were positive for *spvC*, *spvR*, and *bla*_CTX-M_, and only two isolates were positive for *bla*_SHV_, these genes were excluded from the analyses. Unknown serovars or genovars were also excluded. For AMR genes, *bla_TEM_* showed a lower prevalence (7.69%) in serovars Anatum and Derby compared to Typhimurium 1, 4, [5], 12:i:- (83.33%) and Rissen (53.85%) isolates (Figure 3). For virulence genes, serovars London, Anatum, Uganda, and Typhimurium 1, 4, [5], 12:i:- showed lower prevalence (16.67–66.67%) for *avrA*, *mgtC*, and *pipB*, than serovars Derby and Rissen (84.62–100%). Interestingly, the *sseC* gene showed an opposite trend; being more prevalent (91–100%) among serovars Anatum, Uganda, and Typhimurium 1, 4, [5], 12:i:-, but less prevalent in Derby (30.77%) and Rissen (11.54%) (Figure 3).

Binary logistic regression analysis revealed significant variations among *S. enterica* serovars, all relative to serovar Rissen, in the odds of *bla*_TEM_ AMR and several virulence genes, except for *avrA* and *spi4R*. *bla*_TEM_ gene presence was less likely among serovars Anatum and Derby (*p*-value = 0.018; odds ratio = 0.071; 95% CI = 0.008–0.632). For virulence genes, *hilA* was less likely to be detected in only serovar Anatum (*p*-value = 0.023; odds ratio = 0.152; 95% CI = 0.040–0.772). Serovars Anatum, Uganda, Typhimurium 1, 4, [5], 12:i:-, and London isolates were less likely (odds ratio < 1) to carry *mgtC* and *pipB* (*p*-value < 0.05), relative to serovar Rissen, with an acceptable 95% CI. Meanwhile, opposite trends were observed for *sseC*; being more likely in Anatum, Typhimurium 1, 4, [5], 12:i:-, and London, albeit with high odds ratio values and wide 95% CI, which may suggest low precision due to isolate number differences among serovars and between abattoirs and wet markets.

### 3.4. Phenotypic AMR and Associations Among Salmonella enterica Isolates

**Figure 4:**
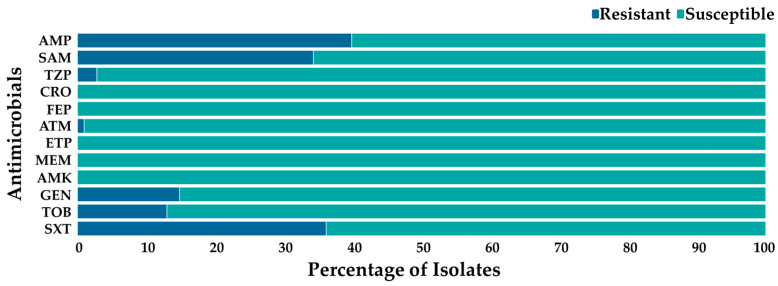
Antimicrobial resistance frequency of *S. enterica* isolates using the VITEK^®^ 2 system.

Antimicrobial susceptibility testing using the automated VITEK^®^ 2 system of known serovars and unestablished genovars revealed resistance primarily to β-lactams, aminoglycosides, and a folate pathway inhibitor. The three highest resistances were from ampicillin (39.81%), trimethoprim/sulfamethoxazole (36.11%), and ampicillin and sulbactam (34.26%), with no resistance to cephalosporins and carbapenems (Figure 4). Most isolates that were resistant to ampicillin were also resistant to ampicillin with β-lactamase inhibitor sulbactam. Multidrug resistance (MDR), which is the resistance to ≥3 antimicrobial classes, was also observed in 14.81% of *S. enterica* isolates with the only MDR pattern involving three antimicrobial classes and five antibiotics, namely ampicillin (β-lactam), ampicillin and sulbactam, gentamicin (aminoglycoside), tobramycin (aminoglycoside), and trimethoprim/sulfamethoxazole (folate pathway inhibitor).

Statistical analysis of phenotypic and genotypic AMR using Fisher’s exact test, specifically with *bla*_TEM_, showed significant associations (*p* < 0.05) between this gene and resistance to β-lactams, ampicillin, as well as ampicillin and sulbactam, but no significant associations (*p* > 0.05) between *bla*_TEM_ and trimethoprim/sulfamethoxazole.

## 4. Discussion

Most molecular studies in the Philippines utilize multiplex PCR of O- and H-antigen-associated genes for *Salmonella* serogrouping and serotyping, which result in putative serovar identification, requiring confirmation with the KWL scheme or WGS. The current study revealed high assignment rates (90%) using the CTS assay. Previous studies using CTS also reported high accuracy (>96%) for commonly occurring serovars with 85–100% concordance to the conventional KWL scheme and WGS. However, CTS falls short with some rare serovars due to database limitations and antigenic phase variations [16,32,33]. Some rare serovars that were discrepant included Adelaide, Arechavaleta, Bracknell, Poona, and Virchow, with some conflicts in Typhimurium 1, 4, [5], 12:i:- and Enteritidis [16,33,34]. However, despite CTS ranking only third with 82.9% correct identification when compared with other commercial molecular-based kits, such as SGSA (microarray), Salm SeroGen (bead-based hybridization), and xMAP (bead-based hybridization), CTS possessed a greater pool of detectable serovars. The current study further supports the accuracy and reliability of the CTS system as a middle-ground for *Salmonella* serotyping, even detecting rare serovar Soerenga, which improves upon subjective and resource-intensive traditional serological testing and is more accessible and cost-effective than WGS for routine surveillance.

Diverse *S. enterica* serovars from different serogroups were observed among isolates in the current study. Serovars Rissen, Derby, Typhimurium (1, 4, [5], 12:i:-), and Heidelberg belong in serogroup B. Uganda, London, Weltevreden, Hvittingfoss, and Mbandaka belong in serogroup C1. Newport and Soerenga belong in serogroup C2–3. Javiana belongs in serogroup D. Anatum belongs in serogroup E. This means that the most dominant serogroup in the current study was B (53.54%), followed by C1 (25.25%), E (13.13%), C2–3 (5.05%), and D (3.03%) in last place. Previous reports in the Philippines also reflect these occurrences. Molecular serotyping of *S. enterica* from retail meats in wet markets in Metro Manila revealed serogroup E, such as Anatum, as the most prevalent [13,18,35]. On the other hand, serogroup B, specifically serovar Typhimurium, was the most prevalent from swine jejunum and tonsil samples in Metro Manila abattoirs [19]. Meanwhile, Calayag et al. [20] reported serogroups E and C1 as the most prevalent among abattoir isolates. In Philippine clinical settings, the most common serovars infecting humans include Typhi, Typhimurium 1, 4, [5], 12:i:-, and Enteritidis, with some frequency of Weltevreden, Anatum, Rissen, and Derby [36,37], suggesting a potential zoonotic threat.

Animal sources, location types, and contamination points may serve as drivers for *S. enterica* serovar diversity. The current study found an overall predominance of serovar Rissen, especially from abattoir swine samples, which may be attributed to their endemicity in swine [38]. Rissen is one of the most common *S. enterica* serovars in Asia and Europe, often implicated in swine [39,40,41,42], attributed to zoonotic transmission, as well as carriage of AMR plasmids [38]. This is further evidenced by an emerging trend reported in China from 1995 to 2019, with a predominant detection of Rissen in humans (63%) compared to foods, animals, or the environment [43]. Following serovar Rissen in prevalence, Derby and Typhimurium 1, 4, [5], 12:i:- have also been documented to be endemic in swine. Both Derby and Typhimurium 1, 4, [5], 12:i:- were among the most common serovars reported in swine from the UK and US [44,45], France [46,47], Europe [48,49], and China [50], with frequent associations to MDR phenotypes. In Europe, *S*. Typhimurium 1, 4, [5], 12:i:- is the third most common serovar in human infections, exhibiting extremely high MDR rates including resistance to last-line antibiotics like colistin [49]. These implications have been attributed to infected but often asymptomatic swine prior to slaughter or to cross-contamination in abattoir settings. As a result, pork ranks third in causing human salmonellosis outbreaks in Europe, with strong epidemiological association and source attribution, following eggs and baked products [48]. Similarly, human infections caused by *S.* Derby, although less severe [46], have also been associated with swine, exhibiting similar genetic and genomic characteristics, as well as AMR profiles [48]. Taken together, these studies suggest that these three serovars are persistent in swine reservoirs, which drives their dominance throughout the swine production chain and presents significant public health implications.

Meanwhile, Anatum was the second most prevalent serovar in the current study, with a higher likelihood in wet markets compared to abattoirs, suggesting a contamination pathway rather than a farm origin. In most literature, serovar Anatum is more commonly associated with calves, cattle, or bovines, as seen in Mexico [51], calves from France [52], and beef from retail markets in Vietnam [53]. It has also been found to be rare in pork meat [54]. Madoroba et al. [55] reported that serovar Anatum was only isolated from hides, but not from carcasses or intestinal contents, of cattle from South Africa, which also suggests external contamination. In Sichuan, China, the most detected serovars among swine farms, abattoirs, and market samples were Derby and Typhimurium, with little to no Anatum detected [56,57]. Despite its higher prevalence and AMR rates in food animals, *S.* Anatum is less frequently implicated in human salmonellosis, although its clinical significance may be emerging, region-specific, and understudied [51,53]. These results suggest that the prevalence of Anatum in the current study may have resulted from post-harvest cross-contaminations, especially during processing in abattoirs and wet markets, rather than persistence in source reservoirs. Nevertheless, this presents food safety risks and the need for abattoir- and market-level interventions to prevent dissemination of *S. enterica* and other foodborne pathogens in the Philippine swine industry.

The current study is also the first to detect the rare serovar Soerenga in the Philippines and in Asia. This finding is noteworthy considering that there is scarce global information about this serovar and a lack of data on its public health or clinical significance. Serovar Soerenga was first isolated and documented by Kauffmann and Bovre in Oslo, Norway [58]. Recently, it was also isolated from a pregnant swine in an intensive farm in La Pampa, Argentina, showing resistance to 17 antimicrobials and possessing numerous virulence genes [59]. This serovar has also been detected, albeit in very low prevalence, from various feedstuffs in Costa Rica [60], in raw and processed broiler chicken feeds, and pelleting equipment from the UK [61], and irrigation water from a country in West Africa [62], suggesting its nature as a sporadic and emerging contaminant. Genomic sequencing of this serovar, isolated from the feces of captive wild birds in Nigeria, has also been recently reported [63]. Although serovar Soerenga is rarely detected, its presence in the local context highlights the importance of continuous surveillance to uncover emerging and rare serovars that may remain undetected and have unestablished public health implications.

β-lactamases are enzymes that confer resistance to clinically significant β-lactam antibiotics, including penicillins, cephalosporins, carbapenems, and monobactams. They are conferred by numerous subtypes of *bla* genes, with *bla*_TEM_, *bla*_SHV_, and *bla*_CTX-M_ among the common class A β-lactamases. Typically, *bla*_TEM_ and *bla*_SHV_ confer resistance to a narrow range of antibiotics, including ampicillins and early-generation cephalosporins. At the same time, *bla*_CTX-M_ is capable of conferring extended-spectrum β-lactamase (ESBL) resistance, including resistance to third-generation cephalosporins and β-lactamase inhibitors [64]. These three *bla* genes are often reported among *Enterobacteriaceae* of human and animal origin and remain a significant public health threat worldwide [65]. The current study showed a prevalence of *bla*_TEM_ exceeding 40% among swine isolates, with a few positives for *bla*_SHV,_ and none for *bla*_CTX-M_. These results were corroborated by the observed phenotypic resistances to ampicillin (39%) and ampicillin-sulbactam (34%). However, no resistance to cephalosporins was observed, although some resistance to aminoglycoside and sulfonamide antibiotics was noted. Similarly, Calayag et al. [21] reported >60% prevalence of *bla*_TEM_ from Metro Manila abattoir samples, showing co-carriage with *qnr* genes among 45% of isolates but only 3% co-carriage with *bla*_CTX-M_, with the highest phenotypic resistance to ampicillin (>70%) and few resistances to cephalosporins (<10%). In the UK, *bla*_TEM_ was the most common AMR gene in *Salmonella* from the environment, humans, swine, poultry, and sheep, but not in cattle, with considerable resistance to ampicillin (>20%) and other non-β-lactam antibiotics [66].

Meanwhile, in poultry and broiler populations, *bla*_CTX-M_ is more commonly reported and has been associated with specific *S. enterica* serovar Infantis throughout Europe and Asia, contributing to high MDR rates and presence of numerous AMR and virulence genes [49,67,68,69,70]. Serovar Infantis has been extensively reported to carry pESI plasmids, which are large (~280 kbp) transmissible megaplasmids that encode numerous antimicrobial, antiseptic, and heavy metal resistance determinants, as well as virulence factors that contribute to bacterial fitness and persistence [71]. In the Philippines, Infantis was also the most detected serovar (>50%) in chicken meats from wet markets, with high ampicillin and cephalosporin resistance, and pESI-like characteristics carrying *bla*_CTX-M-65_, IncFIB(K)_1_Kpn3, and other MDR plasmids such as IncFIA(HI1)_1_HI1 and IncX1_1 [72,73]. Similarly, Madayag et al. [68] reported a prevalence of more than 20% for *bla*_CTX-M_ and only 10% for *bla*_TEM_, with corroborated extended-spectrum cephalosporin resistance found in raw chicken meat at wet markets in Metro Manila. These findings may explain the absence of serovar Infantis and *bla*_CTX-M_ in the current study, as the isolates were obtained from swine samples.

The current study also showed that *bla*_TEM_ was significantly less likely to be present in serovars Anatum and Derby than serovar Rissen, suggesting possible serovar-linked plasmid differences in local contexts. However, the previous literature has shown that, unlike *bla*_CTX-M_, both *bla*_TEM_ and *bla*_SHV_ are more widespread among diverse *Salmonella* serovars, animal reservoirs, and plasmid families. Despite the detection of predominantly *bla*_TEM_, 14% of *S. enterica* isolates in the current study exhibited MDR phenotypes encompassing β-lactams, aminoglycosides, and sulfonamides, which present undetected AMR genes and plasmids. Genomic scale analysis of more than 183,000 *Salmonella* plasmids showed that *bla*_TEM-1_ exhibited high serovar and plasmid entropy, suggesting disseminated carriage in many serovars and plasmid backbones [74]. In another study, IncHI2 plasmids were found to be the most predominant plasmid type among antibiotic-resistant *Salmonella*, housing various *bla* genes such as *bla*_TEM-1_ and bla_OXA-1_, and quinolone resistance genes *qnr* and *aac(6′)-Ib-cr* [75]. In Europe and the US, serovars Typhimurium 1, 4, [5], 12:i:-, Heidelberg, Agona, Derby, and Infantis, from various food animals, have also been reported to carry varying plasmids such as IncF, Inc1, or IncP containing diverse AMR genes, such as *aac*, *aad*, *bla*_TEM_, and *sul* genes, covering different antibiotic classes such as aminoglycosides, sulfonamides, and β-lactams [76,77,78,79]. Similarly, *bla*_SHV_ has been found in broad-host-range plasmids such as IncI1 and IncP [80]. These suggest that the presence of *bla*_TEM_ or *bla*_SHV_ is potentially indicative of carriage of diverse plasmids that require further genome-scale surveillance to uncover further emerging serovars that present concerns for dissemination of virulence, AMR, and MDR genes within the food animal industry.

The current study determined the prevalence of virulence genes within SPIs 1-5 (53–75%) and two plasmid-borne genes, with the most frequent being *hilA*, followed by *avrA*, *mgtC*, *pipB*, *sseC*, and *spi4R.* Additionally, the study found the absence of plasmid-borne *spvC* and *spvR*. SPIs 1-5 are well-documented as the most conserved and genetically stable SPIs across most non-typhoidal *Salmonella* serovars [81,82]. Interestingly, WGS analysis of *Salmonella* Enteritidis and Kentucky isolates from chickens in Iran showed that SPI4 was only present among Enteritidis [83], which may suggest serovar variations and explain the lesser frequency of *spi4R* in the current study. Recent surveillance in local and international contexts also reflects a high prevalence of these SPI 1-5 genes, but with little to no detection of plasmid-borne *spv* genes [23,81,84,85]. However, some studies reported a higher prevalence (30–50%) of *spv* genes or associations with ESBL genes, albeit from different animal reservoirs [86,87]. These suggest variations in virulence gene prevalence and pathogenic potential among *Salmonella* across serovars and reservoirs.

Virulence gene pair associations and co-occurrences in the current study align with previous findings [23,86] and suggest potential cross-talks and complementary functions among SPIs that contribute to *Salmonella* pathogenicity. The *avrA* gene is known to promote intramacrophage survival and modulate the host inflammatory response by reducing Beclin-1 protein expression and stabilizing cell tight junctions [88,89], thereby complementing the functions of *sseC* (SPI2) and *mgtC* (SPI3), which are also involved in intramacrophage survival. While *avrA* and *pipB* are distinct, they are often co-detected and implicated in enteropathogenesis, wherein the former acts on early infection by attenuating inflammation. At the same time, the latter contributes to the intracellular modification of the *Salmonella*-containing vacuole, facilitating its survival [90]. Interestingly, *avrA* is reported not to be regulated by the two SPI1 transcriptional regulator genes, *hilA* and *invF*, which supports the lack of association observed in the current study [91].

Meanwhile, *mgtC* is reported to be essential for long-term intramacrophage survival through Mg^2+^ uptake [92]. While *mgtC* is in SPI3, and *hilA* primarily regulates expression of SPI1 genes during early infection, their co-existence has been reported to enhance systemic infection [93]. This can be attributed to their contribution to the latter intracellular stages of *Salmonella* infection, which facilitates survival and replication [5,90]. The transcriptional activity of *hilA* has also been shown to induce SPI4 and SPI5 gene expression, which are responsible for adhesion and intracellular survival, respectively [94], supporting the associations of *pipB* with *hilA* and *spi4R* observed in the current study. Meanwhile, associations of *sseC* (SPI2) with *mgtC* and *pipB* may be due to their involvement in intramacrophage survival and effector translocation during later stages of the *Salmonella*-containing vacuole, respectively [92,95,96]. Taken together, the observed virulence gene associations in the current study highlight potential interplays within and across SPIs. Although a high prevalence and statistical associations between these genes were detected, it is worth noting that their presence alone does not necessarily signify functionality or cross-talk pathways, which require further expression and mechanistic analyses to elucidate.

The current study showed that *avrA* and *spi4R* were not significantly associated with specific *S. enterica* serovars, in contrast to *hilA*, *mgtC*, *pipB*, and *sseC*. Previous studies have also reported differences. WGS analysis of *S. enterica* from Metro Manila wet markets and abattoirs showed that while *mgtC*, *pipB*, and *sseC* were detected in all serovars, including Anatum, Rissen, and Typhimurium 1, 4, [5], 12:i:-, *avrA* was not present in one Infantis strain [97]. In another study, the presence of *avrA* and *spi4R* varied between two strains of the same serovar, isolated from the same samples, while *hilA*, *mgtC*, and *pipB* were more conserved [98]. Insertions, deletions, and loss or gain of restriction endonuclease cleavage sites may also contribute to variations within SPIs. Amavisit et al. [99] observed that *avrA* (SPI1) was replaced by a 200 bp fragment in serovars Choleraesuis and Ohio. Comparative genomics of serovars Derby and Mbandaka revealed numerous lost or gained genes across SPIs, but notably lacked the supposedly essential *mgtC* gene, unlike serovar Typhimurium [100]. Plasmid-borne virulence genes *spvC* and *spvR* are often required for the complete virulence and systemic infection capability of *Salmonella*. However, these genes are typically present only in a few serovars such as Typhimurium, Enteritidis, and Choleraesuis [101]. Serovar Typhimurium and its monophasic variant 1, 4, [5], 12:i:- have also shown differences with *spvC* presence [102]. Hence, virulence and pathogenicity vary extensively among *Salmonella* serovars, emphasizing the need for molecular surveillance, characterization, and in-depth genomic and transcriptomic analyses to understand pathogenic mechanisms to inform policies and interventions in the food animal industry.

While informative, the current study’s findings have limitations that require cautious interpretations. These include the uneven number of isolates across serovars, which may affect statistical accuracy; a limited and varied number of wet market and abattoir isolates which may cause generalizations and affect statistical accuracy; reliance on presence or absence of genes to infer virulence and antimicrobial resistance, which may not necessarily reflect expression, phenotypic profiles or functional capability; the limited number of virulence genes tested which may impact associations and representations of SPIs; detection of only one antimicrobial resistance gene class (*bla*); lack of plasmid extraction and profiling; and more in-depth workflows and analysis such as whole-genome sequencing to uncover diverse unidentified plasmids.

## 5. Conclusions

The current study provides insights into the diverse *S. enterica* serovars that carry numerous virulence and β-lactam antibiotic resistance genes and exhibit various resistance phenotypes, as observed in swine samples from wet markets and abattoirs in Metro Manila, Philippines. The utility of the CTS assay was demonstrated by >90% classification of *Salmonella* isolates into known serovars, with the most predominant being Rissen, Anatum, Derby, Typhimurium monophasic variant 1, 4, [5], 12:i:-, and Uganda, with their diverse distributions in abattoirs and wet markets, reflecting the potential impacts of source environments and contamination points throughout the swine production and processing chain. The detection of serovar Soerenga in the Philippines and Asia for the first time also highlights the need for further surveillance efforts to capture other rare and emerging serovars, as well as other foodborne pathogens. Genotypic detection of *bla* AMR and virulence genes revealed a high prevalence among *Salmonella* isolates, indicating their resistance and pathogenic potential. SPIs 1-5 virulence genes showed a prevalence of more than 53%, with *hilA* being the most common, followed by *avrA*, *pipB*, *sseC*, and *spi4R*, which reflect. Their widespread distribution and absence of plasmid-borne *spvC* and *spvR*, which are more often associated with a few serovars, is notable. Significant associations between virulence genes suggest possible related or complementary roles in expression and function during *Salmonella* pathogenicity programs that require further elucidation of mechanistic cross-talks. Virulence gene variations were also demonstrated in the current study with significant predictions to specific serovars, except for *avrA* and *spi4R*, which further supports the diverse disease-causing potential and clinical manifestations among *Salmonella* serovars. Lastly, AMR profiling of the *Salmonella* isolates revealed a high prevalence of *bla*_TEM_, a few *bla*_SHV_, and the absence of *bla*_CTX-M,_ which corroborated phenotypic resistance to ampicillin but not to higher-generation cephalosporins. Resistances were also observed to other antimicrobial classes, including aminoglycosides and sulfonamides, with MDR phenotypes. This study highlights the complex and dynamic nature of *Salmonella* contamination, virulence, and resistance among the swine industry in Metro Manila, Philippines. Hence, there is a need for extensive genotypic and phenotypic characterizations of *Salmonella* and other foodborne pathogens, as well as continuous and comprehensive surveillance throughout the production chain, accompanied by stricter antimicrobial regulations and improved food safety and sanitation measures, to ensure both animal and human health.

## Figures and Tables

**Table 1:** Primer sequences, amplicon sizes, and references for PCR assays.

Genes	Primers	Sequences (5′-3′ Direction)	Amplicon Size (bp)	References
Virulence				
*invA* (SPI1)	*invA* ^F^	ACAGTGCTCGTTTACGACCTGAAT	244	[22]
	*invA* ^R^	AGACGACTGGTACTGATCTAT		
*avrA* (SPI1)	*avrA* ^F^	GTTATGGGACGGAACGACATCGG	385	[24]
	*avrA* ^R^	ATTCTGCTTCCCGCCGCC		
*hilA* (SPI1)	*hilA* ^F^	CTGCCGCAGTGTTAAGGATA	497	
	*hilA* ^R^	CTGTCGCCTTAATCGCATCGT		
*sseC* (SPI2)	*sseC* ^F^	TATGGTAGGTGCAGGGGAAG	121	[25]
	*sseC* ^R^	CTCATTCGCCATAGCCATTT		
*mgtC* (SPI3)	*mgtC* ^F^	TGACTATCCAATGCTCCAGTGAAT	655	[26]
	*mgtC* ^R^	ATTTACTGGCCGCTATGCTGTTG		
*spi4R* (SPI4)	*spi4R* ^F^	GATATTTATCAGTCTATAACAGC	1269	[27]
	*spi4R* ^R^	ATTCTCATCCAGATTTGATGTTG		
*pipB* (SPI5)	*pipB* ^F^	TAATGTGCCACATACAGGTAACGC	789	[28]
	*pipB* ^R^	TTCTGGAGGATGTCAACGGGTG		
*spvC* (Plasmid)	*spvC* ^F^	ACTCCTTGCACAACCAAATGCGGA	571	[22]
	*spvC* ^R^	TGTCTTCTGCATTTCGCCACATCA		
*spvR* (Plasmid)	*spvR* ^F^	ATGGATTTCATTAATAAAAAATTA	894	[29]
	*spvR* ^R^	TCAGAAGGTGGACTGTTTCAGTTT		
β-Lactam Antibiotic Resistance				
*bla*_TEM_	*bla*_TEM_ ^F^	TCGCCGCATACACTATTCTCAGAATGA	445	[30]
	*bla*_TEM_ ^R^	ACGCTCACCGGCTCCAGATTTAT		
*bla*_CTX-M_	*bla*_CTX-M_ ^F^	ATGTGCAGYACCAGTAARGTKATGGC	593	
	*bla*_CTX-M_ ^R^	TGGGTRAARTARGTSACCAGAAYCAGCGG		
*bla*_SHV_	*bla*_SHV_ ^F^	ATGCGTTATATTCGCCTGTG	747	
	*bla*_SHV_ ^R^	TGCTTTGTTATTCGGGCCAA		

^F^ Forward primers, ^R^ Reverse primers.

**Table 2:** Singleplex and multiplex PCR protocols for virulence and antimicrobial resistance genes detection.

Gene	ID	D	A	E	C	FE
Virulence						
*invA*	95 °C 2 min	95 °C 30 s	60 °C 30 s	72 °C 30 s	30x	72 °C 5 min
*avrA*, *sseC*, *mgtC*, and *pipB*	94 °C 4 min	94 °C 1 min	58 °C 2 min	72 °C 2 min	35x	72 °C 5 min
*hilA* and *spvR*	95 °C 3 min	95 °C 30 s	50 °C 30 s	72 °C 30 s	35x	72 °C 5 min
*spvC*						
*spi4R*	94 °C 4 min	94 °C 1 min	58 °C 1 min	72 °C 2 min	35x	72 °C 5 min
β-Lactam Antibiotic Resistance						
*bla*_TEM_, *bla*_CTX-M_, and *bla*_SHV_	95 °C 3 min	95 °C 30 s	60 °C 30 s	72 °C 1 min	30x	72 °C 10 min

ID-Initial denaturation, D: Denaturation, A: Annealing, E: Extension, C: Number of cycles, FE: Final extension. Note: Rows indicate separate singleplex or multiplex PCR protocols.

**Table 3:** Virulence gene pair associations among *S. enterica* isolates showing two-sided *p*-value significance under Fisher’s exact test performed in SPSS Build 1.0.0.1447 (IBM).

Virulence Genes Pairs	Two-Sided *p*-Values
*avrA + hilA*	0.208
***avrA*** **+** ***mgtC***	**<0.001**
***avrA*** **+** ***pipB***	**<0.001**
*avrA + spi4R*	0.286
***avrA + sseC***	**<0.001**
***mgtC*** **+** ***hilA***	**<0.001**
***mgtC*** **+** ***pipB***	**<0.001**
*mgtC* + *spi4R*	0.204
***pipB*** **+** ***hilA***	**0.009**
***pipB*** **+** ***spi4R***	**0.026**
*spi4R* + *hilA*	0.182
*sseC* + *hilA*	0.824
***sseC*** **+** ***mgtC***	**0.018**
***sseC + pipB***	**<0.001**
*sseC + spi4R*	0.336

Virulence gene pairs in bold font indicate significant association (*p* < 0.05).

## Data Availability

The original contributions presented in this study are included in the article. Further inquiries can be directed to the corresponding author.
